# Perspectives From Underserved African Americans and Their Health Care Providers on the Development of a Diabetes Self-Management Smartphone App: Qualitative Exploratory Study

**DOI:** 10.2196/18224

**Published:** 2021-02-26

**Authors:** Tai Barber-Gumbs, Ylva Trolle Lagerros, Laura M Sena, Joel Gittelsohn, Larry W Chang, Wayne W Zachary, Pamela J Surkan

**Affiliations:** 1 Program in Public Health Johns Hopkins University Baltimore, MD United States; 2 Department of Medicine Karolinska Institutet Stockholm Sweden; 3 Center for Obesity Academic Specialist Center Stockholm Health Services Stockholm Sweden; 4 Department of International Health Johns Hopkins Bloomberg School of Public Health Baltimore, MD United States; 5 Department of Medicine Johns Hopkins University School of Medicine Baltimore, MD United States; 6 Starship Health Technologies, LLC Fort Washington, PA United States; 7 Social and Behavioral Interventions Program Department of International Health Johns Hopkins Bloomberg School of Public Health Baltimore, MD United States

**Keywords:** diabetes, mHealth, type 2 diabetes mellitus, diabetes self-management, mobile app, mobile phone

## Abstract

**Background:**

Type 2 diabetes mellitus (T2DM) affects approximately 10% of the US population, disproportionately afflicting African Americans. Smartphone apps have emerged as promising tools to improve diabetes self-management, yet little is known about the use of this approach in low-income minority communities.

**Objective:**

The goal of the study was to explore which features of an app were prioritized for people with T2DM in a low-income African American community.

**Methods:**

Between February 2016 and May 2018, we conducted formative qualitative research with 78 participants to explore how a smartphone app could be used to improve diabetes self-management. Information was gathered on desired features, and app mock-ups were presented to receive comments and suggestions of improvements from smartphone users with prediabetes and T2DM, their friends and family members, and health care providers; data were collected from six interactive forums, one focus group, and 15 in-depth interviews. We carried out thematic data analysis using an inductive approach.

**Results:**

All three types of participants reported that difficulty with accessing health care was a main problem and suggested that an app could help address this. Participants also indicated that an app could provide information for diabetes education and self-management. Other suggestions included that the app should allow people with T2DM to log and track diabetes care–related behaviors and receive feedback on their progress in a way that would increase engagement in self-management among persons with T2DM.

**Conclusions:**

We identified educational and tracking smartphone features that can guide development of diabetes self-management apps for a low-income African American population. Considering those features in combination gives rise to opportunities for more advanced support, such as determining self-management recommendations based on data in users’ logs.

## Introduction

As of 2020, an estimated 34.2 million people (10.5%) had diabetes and it was the seventh leading cause of death in the United States [1]. Non-Hispanic African Americans have a notably higher diabetes prevalence (11.7%) compared to non-Hispanic White people (7.5%) [1]. To avoid complications, living with diabetes involves constant self-management, including exercise, healthy eating, glucose monitoring, and adherence to medications [2]. However, low-income African Americans and other minority populations face structural barriers to self-management, such as transportation-related issues, poor health literacy, and limited access to health services [3,4]. A study of almost exclusively Black and Hispanic participants suggested that individuals living with chronic conditions like type 2 diabetes mellitus (T2DM) have been turning toward technology to help with diet, exercise, and weight loss [5].

Research on the use of mobile health (mHealth) strategies to support individuals with chronic diseases, such as T2DM, has shown promising results, particularly with respect to positive lifestyle changes and self-efficacy [6]. Common features of current diabetes apps include encouraging self-management activities via reminders; collecting, storing, and displaying behavioral data on the user’s physical activity, nutritional intake, and medication adherence; offering educational information on diet, nutrition, and lifestyle; and, to a lesser degree, enabling social media connections to other app users. A few make behavioral data available to health care providers, though generally not via the electronic health record [7-12]. Although the effects were small (ie, around a 0.5% change in glycated hemoglobin [HbA_1c_]), a meta-analysis of controlled trials of diabetes apps found that a range of apps significantly reduced HbA_1c_, an indicator of average blood glucose level over time [13].

Minority communities have had limited involvement in the development of mHealth interventions or in the comparative assessment of mHealth apps and their relevance to those communities [5]. Diabetes education that is culturally tailored to African Americans with T2DM can enhance self-management of the condition [14]. However, most diabetes apps on the market are not evidence based [11,15] and likely do not reflect the needs of ethnic, minority populations such as African Americans.

A diabetes app, developed in collaboration with an underserved African American community, could serve as a culturally sensitive and cost-effective tool for diabetes self-management. Thus, we aimed to understand from this community what diabetes app features are perceived to benefit people with T2DM or pre-T2DM for diabetes self-management.

## Methods

### Overview

Between February 2016 and May 2018, we conducted exploratory qualitative research to inform the development of a diabetes management mHealth app, the Diabetes Networking Tool (DNT). Given the wealth of data on social support and its uses in an app, this paper is a companion to a recent separate publication related to desired social support mechanisms in an app [16].

### Setting

The study took place in Southwest Baltimore, a low-income neighborhood where almost three-fourths of residents were African American [17]. In 2017, the median household income of the neighborhood was only slightly over half (ie, US $24,946) that of Baltimore City overall (ie, US $41,819). In 2017, the age-adjusted mortality rate for diabetes was 4.4 deaths per 10,000 in Southwest Baltimore, which can be compared to Baltimore City’s average rate of 3.0 deaths per 10,000 [17].

### Recruitment

Participants were initially recruited at residential buildings, a farmer’s market, and a supermarket. Subsequent recruitment was done through snowball sampling. We used stratified, purposive sampling to achieve a distribution by gender and disease status (ie, prediabetes and T2DM versus close friends and family). Providers were recruited from health care facilities in the study area or we recruited those serving similar low-income populations. Nonproviders were incentivized with gift cards of US $40 or US $50, depending on the data collection activity.

The Johns Hopkins Bloomberg School of Public Health Institutional Review Board approved data collection. All participants provided oral informed consent, which included consent that deidentified data might be shared for research purposes.

### Study Sample

A total of 78 people participated in this study. Inclusion criteria for nonproviders were being English-speaking adults who self-identified as having either prediabetes or T2DM or being a friend or family member of someone with prediabetes or T2DM, residence in the study community, and owning a smartphone. We included health care providers who served predominantly low-income African Americans with prediabetes or T2DM. Out of the 78 participants, 28 (36%) self-identified as having prediabetes or T2DM, 30 (38%) self-identified as being a friend or family member of someone with T2DM, and 20 (26%) were health care providers (eg, diabetes educators, pharmacists, nurses, and physicians). Upon sign-in at the data collection activities, participants with T2DM and family or friends of persons with T2DM were asked to self-report their race, whether they identified as Hispanic or not, and whether others in their families had T2DM.

### Data Collection

Data were collected in three phases. In Phase I, we held a series of 2- to 3-hour-long in-person interactive forums and interviews with people with T2DM and family or friends, one focus group, and multiple interviews with health care providers. The interactive forums and the focus group discussions were facilitated by public health graduate students, an anthropologist, or a public health faculty member who taught or was trained in qualitative research methods. Forums generally were larger than focus groups and used more interactive aides to guide the discussion, though the topics covered in the guide were very similar. Facilitators used semistructured interview guides and engaged participants in the creative process by asking them to make suggestions, offer ideas, and build on suggestions of other participants. Forums were audio-recorded and transcribed verbatim for analysis. Guides for the semistructured interviews also covered similar content, with the main difference being that they were longer and included more probes. Interviews were mainly carried out when it was logistically difficult to schedule a group session.

**Figure 1:**
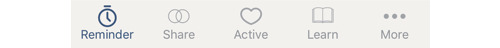
Main navigation panel within the app.

**Figure 2:**
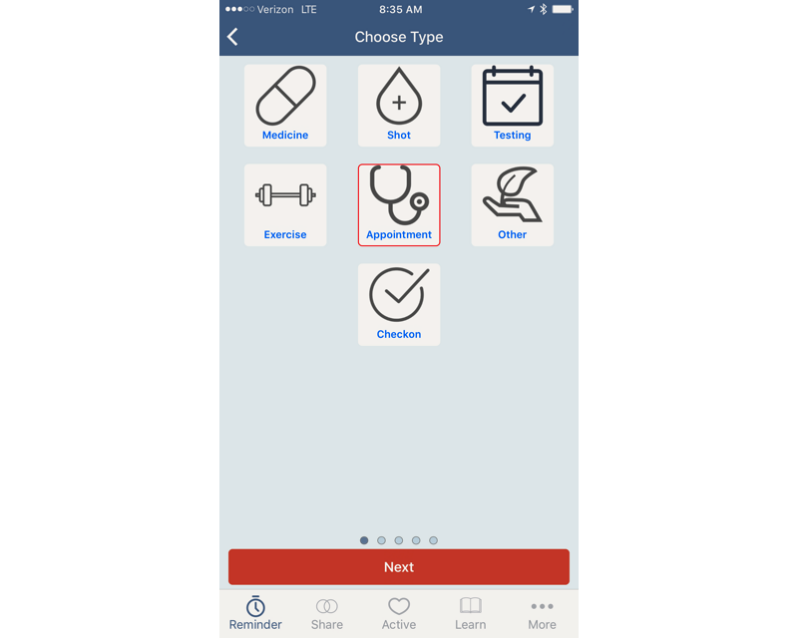
Initial detail screen when “Reminder” is selected from the navigation panel of the app.

**Figure 3:**
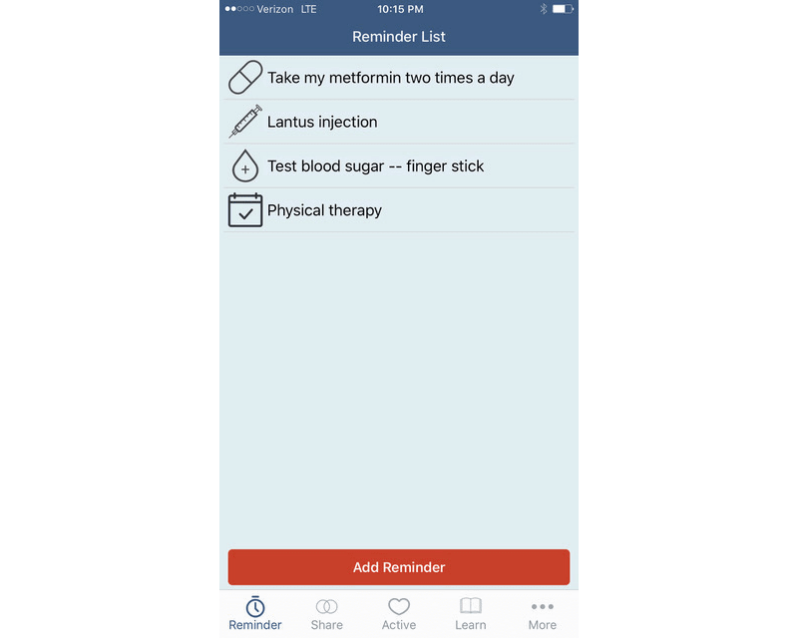
Initial screen when in the process of setting a new reminder in the app.

From Phase I, we analyzed participants’ opinions on design concepts for a smartphone app [18]. In Phase II, we explored reactions of community members and providers to those design concepts through one community forum with people with prediabetes or T2DM and family or friends, as well as through interviews with providers, due to difficulties in organizing providers into groups. Using Phase II inputs, a revised design and prototype were created. Figures 1-3 provide prototype screen examples. Phase III data collection involved two forums that covered community members’ perceived usefulness, usability, and learnability of the prototype, supplemented by interviews with 4 participants with T2DM.

### App Prototype

The results from the Phase I and Phase II data analyses were used to develop design concepts. Afterwards, a preliminary prototype of an app was developed. We presented static mock-ups of specific screens in this hypothetical app to community members in Phase II to receive comments, learn of perceived problems, and get suggestions to improve the design.

Revised designs were then animated to simulate dynamic sequences of app use. We presented this final interactive prototype app to users in Phase III for qualitative discussion and performed a quantitative assessment using the widely used System Usability Scale (SUS) [19,20]. This instrument uses a 10-item Likert scale to measure perceived usability and perceived learnability of a software interface.

### Qualitative Data Analysis

Based on the community-generated ideas, we carried out thematic data analysis using an inductive approach to explore ideas about app use in diabetes self-management [21]. An inductive approach entails seeking patterns in the data without using a predetermined theory. Steps in our thematic analysis included the following: (1) reading and rereading transcripts to become familiar with that data, (2) generating initial codes, (3) looking for themes among codes, (4) reviewing these themes, and (5) defining and naming them [21]. Two researchers coded the data (LS and PS). Initial coding was done with the aid of the qualitative software ATLAS-ti, version 7 (Scientific Software Development GmbH), followed sequentially with additional code refinement by another author to further capture the richness of the data (TBG). Memos were used throughout the analysis to facilitate theme development [22]. Data collection and transcription occurred concurrently, and transcripts were reviewed to elicit themes using the constant comparative method [23].

## Results

### Overview

Table 1 displays participants’ demographic characteristics by type and data collection event. Community participants were adults who were evenly split between those diagnosed with T2DM or prediabetes (28/57, 49%) and family or friends (30/57, 53%). The majority of community participants (37/58, 64%) and of all participants (47/78, 60%) were female. A total of 4 people participated in forums across two different phases. No participants self-identified as Hispanic. Table 1 shows the number of unique individuals who participated as persons with prediabetes or T2DM or as family or friends. 

**Table 1:** Participant characteristics and data collection events.

Characteristic	Prediabetes or T2DM^a^ and friend or family participants (n=58), n (%)					Provider participants (n=20), n (%)					Total participants (N=78)
	Initial forums (four)	Preusability forums (two)	In-depth interviews	Subtotal	Initial FGD^b^ (one)		In-depth interviews	Paired interview (one)	Subtotal		
Total by event	41 (71)	13 (22)	4 (7)	58 (100)	9 (45)		9 (45)	2 (10)	20 (100)	78 (100)	
Gender: male	15 (26)	5 (9)	1 (2)	21 (36)	5 (25)		4 (20)	1 (5)	10 (50)	31 (40)	
Race: African American	40 (69)	13 (22)	4 (7)	57 (98)	5 (25)		4 (20)	1 (5)	10 (50)	31 (40)	
T2DM status: prediabetes or T2DM^c^	15 (26)	10 (18)	3 (5)	28 (49)	N/A^d^		N/A	N/A	N/A	N/A	

^a^T2DM: type 2 diabetes mellitus.

^b^FGD: focus group discussion.

^c^One person in the forums did not indicate their T2DM or prediabetes status and was not counted here as having T2DM or prediabetes (n=57).

^d^N/A: not applicable; T2DM status was not applicable to providers.

We identified three main themes that identify desired goals that a smartphone app could help achieve, including help in (1) getting access to health resources, (2) delivering patient education about T2DM, and (3) supporting the diabetes self-management process.

### Finding and Accessing Health Resources

All three types of participants reported that accessing care, medication, and testing resources were major barriers to self-managing diabetes. A provider explained the following:

They're [people with diabetes] eligible mostly for Medicaid, but for whatever reason they don't have insurance and they don't have funds for their medication. We have a couple of clients who have used the ER [emergency department] for getting their medication...We also see a lot of times people are running into challenges with getting supplies they need—like the glucose monitor, the lancets, the strips.

Several participants described how an app could make it easier to find free or discounted medication and medical supplies. For example, an individual with T2DM suggested the following:

You could talk to the app like, “Hey I lost my medicine, I don’t have the insurance to pay for it. Is there some place I can go to get a free meter, a place for free testing strips, a pharmacy that may give out free insulins or medications?”

An app could also function as an alternative to computers. A patient explained how in the following quote:

People [in the study community] don't have access to the computer where they can actually go online and find [the discounts] and then end up waiting until the last minute or say, “Well, I can skip this,” and you can't skip a dose. I tried it, believe me. I have been diabetic for over 20 years, and it’s in my family. I've seen in my family a lot of missing toes.

An app could inform people with T2DM and/or their family or friend caregivers about affordable resources, such as free or low-cost medications, testing supplies, and/or healthy foods. Participants agreed that the community does provide resources, but that people with T2DM are not aware that they are available. One provider explained, “It is a city, so the resources are there...it's just being aware of the resources.” A patient stated, “If I don't know what’s going on, nine times out of ten, I am not going to come out [to access resources] if I don't know.”

Some participants expressed how an app could help people with T2DM find free resources to help with self-management. Others thought user posts on social media could help address such resource-finding problems. For instance, “If I wanted to post something, what would it be? I'd post locations for farmer's markets, community gardens.” One provider suggested the following:

We tell our patients to go to—it’s called Baltimore Free Farm on Arch Street—and every Wednesday they literally give away food and there’s so many people when they come to their appointment and they say, “I don’t have money to eat anything let alone something healthy.” So, I think if a careful search is done of those resources and that’s included on the app, that would be tremendous.

### Need for More Information About Diabetes

Participants agreed that more information was desired about prediabetes and T2DM self-management and that an app could provide accurate T2DM information in lay terms. T2DM participants expressed how lack of knowledge discouraged them from seeking medical care:

I really don't know nothing about diabetes. So, I be like hesitant to go back to the doctor, because I don't want to start taking stuff that I don't know nothing about.

Some participants with T2DM discussed a need for an app to help fill the information gap:

I think if it could be kinda like Siri, if you can talk to it and ask it questions, then that would probably be good too...if you could ask the app questions like “What happens if I don’t take my insulin for two days?”

Nonetheless, because of concerns about false information being spread on an app platform, participants suggested this information could be based on reliable sources (eg, the State Department of Health and Mental Hygiene).

### Active Support for Diabetes Self-Management Tasks and Activities

#### Overview

The third area in which participants suggested an app could help was in self-management itself, by structuring and automating some repeated care tasks in T2DM (eg, taking medications and tracking blood glucose). Suggestions from all three types of participants fell into two interrelated areas: (1) app support for recording and tracking behaviors and testing data relevant to T2DM self-management and (2) a higher level of structure for self-management, to make it easier to adhere to and keep motivated.

#### Logging and Tracking Information

Participants expressed that if people with T2DM could track their progress and receive encouragement, then they would be likely to follow through with diabetes self-care plans. Some participants suggested that tracking could allow people to input their diabetes care information, such as HbA_1c_ levels, and to set reminders for care activities, such as when to exercise. A person with T2DM explained as follows:

That’s how we know who’s cheating, and who’s not checking their sugars and things like that; in keeping a record, you can see when I did my last finger stick, so I know what it was.

Likewise, several providers expressed frustration about how patients forget their glucose monitors, yet they noted how most people with T2DM bring their phones to the doctor’s office. Providers suggested that if patients registered health data on their phones, it would be easier to track what they were doing. A provider explained as follows:

In my last job...they would bring their glucose monitor and it would just link to our computer and we would be able to download all of their data and it would give us a graph and we would be able to see trends and patterns and whether they are truly checking or not...I would like that information

on an app

By tracking self-management behaviors, people with T2DM could be held accountable to their health care providers. Friends and family members were also interested in having access to health information stored on an app so that additional support could be given. A friend or family member of a person with T2DM described this view as follows:

[The person with diabetes] can share it with me. It's another way you can help them, so when you go to the doctor and you have three months [of tracking], and they are able to send this through the app to the doctor’s office and they see this, then they can know what to do to change your regimen.

In addition to monitoring progress, participants wanted an app that could remind people with T2DM to stay on track with their diabetes care plan. A participant with T2DM explained as follows: “Just something to keep the diabetes in the front of the brain instead of the back of the brain.” Moreover, an app that has a reminder function could help people with T2DM remember their appointments or to refill medications. A provider pointed out the following: “We have a really high no-show rate here in West Baltimore, so a reminder for appointments is good.”

#### Encourage Engagement With Self-Management

For persons with T2DM, self-management is a process that requires constant attention. Difficulties with diabetes self-management often lead to psychological distress and decreased adherence [24,25]. Participants suggested that, for the sake of further encouraging people with T2DM to stick with their care plans, an app should incorporate a goal-setting mechanism based on feedback and reward. By setting diabetes-related goals, a friend or family member explained that someone with T2DM could more easily achieve them:

The most important things would be to set a goal for them at the beginning—maybe weight loss, maybe HbA_1c_—so they can follow it and figure out how to get to that goal.

Then, by providing feedback on those goals via an app, patients would know the steps needed to stay on track with their health management plan. Finally, by setting goals and tracking one’s progress toward them, enabled by the data recording and tracking function, an app could help evaluate progress, ideally in conjunction with a health care provider, and give feedback. A provider recommended the following:

The app should be able to analyze the data that the patient is putting in there and give them some feedback. “Oh, gee...your blood sugar has been in goal for the last five days—congratulations!”

This goal-setting, tracking, and feedback structure would enable positive feedback to encourage a person with T2DM to persist with a self-management plan and pinpoint specific problems to work on. Participants suggested that an app could provide, in addition to praise and focused constructive feedback, tangible diabetes-related rewards for people who are doing well. A provider proposed the following:

Maybe if they lose five pounds, they can get a discount on their gym membership for a month, or if they keep their blood sugar in control for two weeks, then maybe they get a week’s supply of fresh vegetables from the farmers’ market...Really, the ultimate reward is going to be that they preserve their health.

### Translation to App Design

Our qualitative data analysis then led to the design of a preliminary prototype app with the following user functions: (1) setting reminders to engage in self-management behaviors (Reminder), (2) sharing information with other users of the app (Share), (3) finding other persons to engage in physical activities as part of T2DM self-management (Active), and (4) learning more about T2DM and related areas (Learn). The navigation design for the app was realized as a navigation panel of four glyphs at the bottom of the screen that corresponded to these four functions, as shown in Figure 1. The navigation panel also included a *More* glyph that could be used to navigate to a group of settings, as shown in Figure 1. Details on the process of functional and navigation design can be found in Zachary et al [18].

The *Reminder* and *Active* functions correspond directly to one of three themes discussed above, specifically “supporting the diabetes self-management process through the app.” The *Reminder* function allows users to create reminders for the self-management activities, while the *Active* function connects users to a local social network of other app users in order to find others to participate, some with whom the user could plan regular physical activity. The *Learn* function was based on the theme of “delivering patient education about T2DM.” It takes the user to lists of curated information from health experts, organized into categories of information needs that repeatedly arose in the forums (eg, information on diabetes, nutrition, exercise, and medications). The *Share* function provides functionality for sharing information with other local app users. One key purpose of this function is to share information on access to local health resources and opportunities, thus addressing the theme of “getting access to health resources.”

A more detailed example of one function (ie, *Reminder*) begins in Figure 2. It shows the initial page of the interaction once *Reminder* is selected; the user’s list of current reminders is shown, as is an option to create a new one. The user can tap an existing reminder to view or change it, or they can tap *Add Reminder* at the bottom of the screen. This takes the user to a palate of glyphs representing different self-management activities, as shown in Figure 3. From there, the user can select the type of reminder and begin the process of setting the details of that reminder.

### App Usability Assessments

We collected data on the prototype app’s perceived usability through two preusability forums (see Table 1). In each forum, participants were introduced to the DNT concept and exposed to the DNT prototype app via an interactive walk-through of the app. A facilitated discussion followed, which covered the prototype, its perceived match to its purpose, its usability and perceived strengths, its weaknesses, and possible improvements. SUS data were analyzed numerically using algorithms defined by Sauro [20]. Out of 13 participants, 12 (92%) completed surveys were returned, and 2 surveys were discarded because the instructions were not followed. The mean SUS score of the usable surveys was 85.5 (SD 22.5), placing the DNT app above the 90^th^ percentile of all systems assessed with the SUS. While this score was very high, the sample (n=10) was small (ie, N≥12 is recommended for SUS analysis) [26] and the variability was high (SD 22.5). Also, participants did not directly interact with the app but observed its use on the user’s phone. Because the participants did not physically interact with the app, we use the term *preusability forum*, rather than usability forum. The high mean score suggests that app functionality and interface design were consistent with the needs identified.

## Discussion

### Principal Findings

In soliciting perspectives in a high-risk community from people with prediabetes and T2DM, as well as from formal and informal caregivers, we found that an app was desired that could (1) address some problems with access to health resources, (2) provide patient education on diabetes and risk factors, and (3) actively support self-management through tracking and encourage long-term adherence to self-care plans.

While some diabetes apps link patients to health resources and providers [27,28], recent comparative reviews did not include addressing financial barriers to diabetes self-management as a comparison criterion [7-11,29]. Moreover, while many apps focus on tracking of self-management adherence, the comparative review articles also did not cover comparisons of features intended to improve long-term user engagement with the app that would be required for effective long-term tracking. These absences suggest that such features were generally lacking.

Low-income families experiencing stress are less likely to be confident of receiving health care and less likely to receive it regardless of insurance coverage [30]. The problem of limited access to health care for low-income families is substantial in the United States. This could be attributable to the complexity of insurance coverage and the need for out-of-pocket funds to meet co-pays [31,32]. This complexity may explain an inconsistency in our data. The data suggested that participants could perceive health services as being available (eg, reporting having insurance through the publicly funded Medicare and/or Medicaid programs), while also reporting not having access to medications. Medicaid, which insures eligible low-income persons, and Medicare, which insures all persons over 65 and eligible disabled persons, have prescription drug components. However, those programs do not cover the full cost of many medications. Thus, they can levy substantial out-of-pocket costs for patients with chronic illness, such as T2DM patients, who require medications year-round [33,34].

A second inconsistency in our data is less easily explained. Participants reported having a smartphone but not being able to access websites with prescription discounts. It is possible that the participants did not know that they could access those websites from their phones or that their cell connections lacked the bandwidth to easily view those websites. Both would be interesting subjects for further research.

While not able to solve the more structural complex problems of lack of insurance and problems with access, our results suggest that an app with a function that would allow community members to post and share information about discounted or free medication and testing supplies in their area would be useful. Given the existence of primary care interventions where social workers check for social and welfare programs that could cover benefits that people with T2DM are not aware of [35,36], an app including this feature would fulfill this need at a lower cost.

This research points to another underexplored way that a diabetes app could benefit the community—by presenting curated information about T2DM so that people recognize and understand how to manage symptoms. Low-income, racial and ethnic minority populations underestimate their chance of developing diseases, which is a risk factor for not seeking regular health care [37]. A US study with a 77% non-White sample found that knowledge gaps were pervasive, yet knowledge about diabetes risk was a motivating factor for better management [38]. This underscores the importance of finding ways to disseminate information about T2DM to these populations.

To our knowledge, research has not examined how effective the consumption of health information among people with diabetes who use mHealth apps has been for self-management; however, apps may be superior to other forms of communication (eg, computers and books) for their convenience, mobility, and timely access to information [39]. There has been rapid adoption of smartphone technology in the United States, even among older and poorer segments of the population [40]. Given this, by making information about chronic disease easily accessible, an app might enable people with T2DM to better navigate diabetes management.

This research indicates interest in app features that focus on maintaining the long-term engagement of people with T2DM in self-management. Although long-term engagement is the ultimate goal, Kitsiou et al concluded in their review that they could only comment on short-term diabetes app studies, due to the lack of studies with long-term follow-up [41].

Regarding participants’ desires for logging and tracking features, two reviews of 181 and 143 diabetes management apps for a medication reminder feature showed that only 56% and 58% of apps had such a function, respectively [42,43], in spite of the fact that those that did had characteristics identified as likely to be effective [43]. Moreover, logging and tracking are typically applied only to a few aspects of self-management, with logging of nutritional intake and blood-glucose testing being more common, and logging of medication adherence being the least common [12]. This point is particularly relevant given challenges in self-management because of residents’ low-income and minority status. Participants valued positive feedback and encouragement that could be enabled by data recording and tracking and automated data analysis, suggesting these two features may be helpful to support self-management. This may be particularly important, because participants often reported not having a consistent primary care provider over time and/or moving among several health systems, meaning that their electronic patient records would likely be incomplete and spread across many different computer systems. In such cases, the self-management log created by the user’s app may be the only longitudinal data available to the provider for that patient.

One more enhancement suggested by participants was to get feedback from providers using tracked data [5]. An analysis of existing diabetes apps showed that improvements in HbA_1c_ levels, in conjunction with diabetes app use, were highly related to feedback from health care providers [13]. For example, dietary logs in combination with medication adherence, glucose testing, and physical activity logs could anticipate situations that might lead to degraded glucose management and alert the app user to take preventive actions.

Gamified smartphone apps fulfill a psychological need for satisfaction and feelings of accomplishment [44-46]. Health gamification can be applicable to an array of health conditions, including diabetes [46-48], and could directly extend from the engagement-enhancement features envisioned by our study participants. Gamification can lead to greater engagement and more persistent use. Positive feedback, driven by the app’s analysis of recorded and tracked data, can trigger more gamified feedback, such as earning badges and/or setting up challenges with others. Such features can encourage people to persist in self-management by making it fun.

### Strengths and Limitations

Multiple perspectives from different types of participants enabled triangulation of the findings. Another study strength was the participatory nature of data collection that incorporated voices of people traditionally overlooked in app development [5].

A study limitation was that most participants with prediabetes and T2DM were African American women, limiting the transferability of these findings. We lacked information on participant age and socioeconomic status, though our impression is that most participants with T2DM were in midlife or older and that they were low-income persons, as they all resided in a disadvantaged neighborhood. Furthermore, unless identifiable by the quote itself based on the audio recordings, we could not always distinguish participants with prediabetes and T2DM versus friends and family.

Finally, the interactive app prototype developed as part of this research did not, and could not, address all the functionality suggested because of the limited exploratory scope of the project. Specifically, it did not attempt to address more complex issues, such as gamification and longitudinal analysis of logged self-management data. Thus, while we identified community-driven ideas for diabetes self-management app features, the feasibility of implementing these features still needs further study.

### Conclusions

In conclusion, despite the proliferation of diabetes mHealth apps [49], a dearth of information exists concerning the usage needs of these apps from African Americans with T2DM and the people who help them with disease management. Some needs uncovered in our study have been relatively uncommonly reported in the literature, such as lack of awareness of available social and welfare programs, of affordable health insurance, and of available, affordable, and local sources of healthy foods. Other needs identified correlate with those of T2DM patients in general (eg, support for self-management, encouragement, and engagement). App features should facilitate addressing these needs and consider incorporating, for example, tracking and gamification features. Future research may extend these findings and assess the feasibility of, and test apps with, these features.

## Abbreviations

DNT: Diabetes Networking Tool
ER: emergency department
HbA_1c_: glycated hemoglobin
mHealth: mobile health
SUS: System Usability Scale
T2DM: type 2 diabetes mellitus
